# SOCIAL PARTICIPATION, LONELINESS, AND CIRCADIAN RHYTHMS OF MIDDLE-AGED AND OLDER ADULTS IN TAIWAN

**DOI:** 10.1093/geroni/igad104.2735

**Published:** 2023-12-21

## Abstract

Loneliness among middle-aged and older adults is a growing public health concern in the aging society. While social participation is known as a protective factor against loneliness, not all older adults enjoy social participation. The purpose of this study is to investigate the social participation and loneliness characteristics in communities and their association with the demographic data, health-related variables, objective sleep parameters and rest-activity circadian rhythms with a sample of 242 Taiwan community-dwelling middle-aged and older adults aged 45 to 89 years. Loneliness and social participation levels were grouped in different clusters via Two-Step Cluster Analysis. Subsequently, Chi-square test and ANOVA were performed to identify the differences between clusters with relevant variables. Results showed that five social participation-loneliness clusters were grouped: Average(40.1%), Active-Lonely(3.8%), Not Active-Lonely(13.1%),Active -Not Lonely(5.9%),Not Active -Not Lonely(37.1%). Age, employment status, property management, self-perceived health, depression, life satisfaction and relative amplitude were related to clusters. The Active-Not Lonely cluster was older, had a higher retirement percentage, and higher life satisfaction. Compared with the Not Active-Lonely cluster, the Not Active -Not Lonely cluster had more power to manage their property, felt healthier and less depressive. The Active-Lonely cluster had a high percentage of unemployment and felt unhealthier. The Not Active-Lonely cluster had the lowest relative amplitude, which meant they had weaker rest-activity circadian rhythms. Objective sleep parameters were no difference between each cluster. These findings provide the potential benefits of developing appropriate social care programs to reduce loneliness for middle-aged and older adults via objective data.

